# Supplementary material for: Let’s talk about race: changing the conversations around race in academia
Source: Commun Biol. 2021 Aug 5;4:902. doi: 10.1038/s42003-021-02409-2 (PMC8342502; doi:10.1038/s42003-021-02409-2)
Supplement: Supplementary file 1 — Supplementary Information [file 42003_2021_2409_MOESM1_ESM.pdf]

Supplementary Information for

Let's Talk About Race: Changing the Conversations Around Race in Academia

Jasmine M. Miller-Kleinhenz[1], Alexandra B. Kuzmishin Nagy[2], Ania A. Majewska[3], Adeola O. Adebayo Michael[4], Saman M. Najmi[2], Karena H. Nguyen[3], Robert E. Van Sciver[5], Ida T. Fonkoue[6]

Affiliations

[1]Department of Epidemiology, Rollins School of Public Health, Emory University, Atlanta, GA, 30322, USA

[2]Department of Biochemistry, School of Medicine, Emory University, Atlanta, GA, 30322, USA

[3]Department of Biology, College of Arts and Sciences, Emory University, Atlanta, GA, 30322, USA

[4]Department of Pediatrics, School of Medicine, Emory University, Atlanta, GA, 30322, USA

[5]Department of Human Genetics, School of Medicine, Emory University, Atlanta, GA, 30322, USA

[6]Renal Division, Department of Medicine, Emory University School of Medicine, Atlanta, GA, USA; Research Service Line, Atlanta Veterans Affairs Health Care System (VAHCS), Decatur, GA, USA.

Corresponding Author*: Jasmine Miller-Kleinhenz, Department of Epidemiology, Rollins School of Public Health, Emory University, Atlanta, GA, USA, jmill37@emory.edu

This PDF file includes:

Eight supplementary notes for discussions on race and social justice along with learning objectives and example materials:

> Supplementary Note 1. Discussion on "Becoming comfortable having uncomfortable discussions surrounding race and social justice within our own group"
>
> Supplementary Note 2. Discussion on "Race as a construct - The history of anti-Black racism in the U.S."

Supplementary Note 3. Discussion on “Having courageous conversations (Braided narrative)”

Supplementary Note 4. Discussion on “Evidence-based practices for mentoring and supporting Black, Indigenous, and people of color (BIPOC) in STEM”

Supplementary Note 5. Discussion on “Investigating microaggressions and macroaggressions as well as their impact in the classroom and beyond”

Supplementary Note 6. Discussion on “Implementing social justice practices”

Supplementary Note 7. Discussion on “Recognizing, alleviating and dismantling local systems of oppression”

Supplementary Note 8. Discussion on “Fostering anti-racist behavior among our students, academic peers, and community organizations”

References for teaching materials

Supplementary Note 1. Discussion on “Becoming comfortable having uncomfortable discussions surrounding race and social justice within our own group”

Major Objective: To practice having conversations on race and social justice as a scientist

Learning Objectives:

1. Discuss how people may take comfort in their own privilege and how that influences their ability to discuss race, social justice, and action
2. Define intersectionality, identify examples of intersectionality, and connect this concept to conversations of race
3. Examine and describe how language and semantics are important in these conversations
4. Practice engaging in conversations about race

Supplementary Table 1. Pre-class assignment and lesson plan with time dedicated to each activity along with resources and references for Discussion 1.

<table>
<tr><td colspan="3">Pre-Class Assignment:</td></tr>
<tr><td colspan="3">1. Write a list of your various identities (some examples: race, gender, sex, age, culture, mother, brother, etc.).
2. Bold the identities that are privileged in The United States.
3. <u>Underline</u> the identities that are met with barriers in The United States.
4. Highlight your identities that intersect (some examples: culture and religion, feminist and lesbian, woman and heterosexual; note that these intersections can be subjective and are based on your own experiences and perceptions). We will discuss our responses and observations in class</td></tr>
<tr><td colspan="3">Lesson Plan</td></tr>
<tr><td>Time</td><td>Activity/Topic</td><td>Resources/References</td></tr>
<tr><td>20 min
(~3 min/person)</td><td>Pre-class assignment discussion</td><td>1. Ask everyone to share their identities (those that they are comfortable sharing).
2. For the identities that intersect, ask them to describe how they intersect.
3. Ask if the identities met with barriers would be different in different contexts (in a different country, in a different field)
4. Define intersectionality
5. Point out any differences in language/semantics
6. Emphasize the purpose of this exercise is to</td></tr>
</table>

		understand:<br>a. People make different connections<br>b. People have different experiences<br>c. Recognizing where we all come from helps inform our interactions with each other<br>*Note to Facilitators: The goal with this activity was to become aware of our own privileges and the contexts in which we face barriers. We centered the conversation around building empathy to see beyond our own perspectives to engage respectfully with others.*
10 min	Discuss your own identity	1. Watch Video: Your Reality Might Not be Mine[1] https://youtu.be/SYytiQmXNTc (Stop video at 3:44)<br>*Note to Facilitators: This video is a TEDx Talk by Dr. Poppy Crum discussing how no person experiences the same reality, or world, in the same way because how people respond to us and how we respond to others is often influenced by our different identities.*<br>2. Watch Video: How to Have a Voice…[2] https://youtu.be/IF--2vGj7Tg (Stop video at 4:48)<br>*Note to Facilitators: This video is a TEDx Talk by Dr. Amdanda Kemp discussing how to meaningfully and respectfully engage in conversations about race, particularly in regards to advocating for one's self and others.*<br>3. Watch Video: "Having the Uncomfortable Conversations" Video<br>*Note to Facilitators: This video is no longer available on YouTube. The video featured a strategy - recognize, interrupt, and repair - to address racism, sexism, etc. as it arises in conversation.*<br>4. Discuss strategies to engaging in uncomfortable conversations, including:<br>a. Recognize<br>b. Interrupt<br>c. Repair<br>5. As a class, brainstorm a rubric for having these conversations
~10 min	Discuss potential confrontational encounters	1. What makes a conversation uncomfortable? Does it include...<br>a. Microaggressions?

		b. Harassment?<br>c. A genuine conversation to learn more, but not all the participants have identities that allow them to be empathetic. (What are the semantics at play? Are apologies genuine, or are they along the lines of "Sorry, but" or "Sorry you were offended"?)<br>2. Where might these conversations occur?
25 min	Practice engaging in conversations about race in breakout groups/rooms	Practice Scenarios:<br>*Note to Facilitators: For each of the following scenarios, there was a corresponding video wherein a Conflict Management and Intercultural Relations Specialist demonstrated different ways to recognize, interrupt, and repair the misconceptions and harmful biases associated with each practice scenario. These videos are no longer available on YouTube.*<br>● "I don't think Joann got this job because she's qualified, I think she got it because she's Black."<br>"He's Black. He must be good at basketball. Let's pick him for our team so we can win."<br>● "If students are going to succeed in my class, they need to speak English."<br><br>1. Present each group with a scenario.<br>2. Ask:<br>○ What is your feeling about that statement when you hear it?<br>○ If you heard that statement, how would you interrupt it? What could/would you say? Would you say anything?<br>○ How can you repair or shift the conversation?<br>3. Ask the class to watch the associated video to see how Nicole (Conflict Management and Intercultural Relations Specialist) navigates these conversations.<br>4. Compare your responses to hers.

Additional videos and practice scenarios for analysis:

- Video and News Article: Birding in Central Park[3]
  https://www.cnn.com/2020/05/26/us/central-park-video-dog-video-african-american-trnd/index.html

- Video: Cultural Differences in a Canadian Workplace[4]
  https://youtu.be/5-3gbex09tk
- Video: Language and Accents[5]
  https://youtu.be/29bobQDLrBA
- Video: Religion in the Workplace[6]
  https://youtu.be/foFdqmdiaR0
- Video: Actively Engaging Diverse Backgrounds in the workplace[7]
  https://youtu.be/6sbNk4cEYbY

Supplementary Note 2. Discussion on “Race as a construct - The history of anti-Black racism in the U.S.”

Major objective: Deepen our understanding of race as a construct: perceptions on race throughout history and the nature of anti-Black racism in the U.S.

Learning Objectives:

1. Describe African American history, including milestones and lingering systems of anti-black racism in the United States.
2. Understand the African Diaspora and how the trans-atlantic slave trade cemented race as a construct in the United States
3. Understand how BIPOC contributed to the labor movement and how this contribution disproportionately benefited white americans.

Supplementary Table 2. Pre-class assignment and lesson plan with time dedicated to each activity along with resources and references for Discussion 2.

**Pre-class Assignment**		
1. Choose an article from *The 1619 Project* and be ready to summarize to the group.[8]<br>a. https://www.nytimes.com/interactive/201w9/08/14/magazine/1619-america-slavery.html<br>2. Watch *The Lie that Underpins All Injustices Facing Black Americans*[9]<br>a. https://www.youtube.com/watch?v=I205IQw6ns8<br>3. Choose a Black scientist s/hero and come ready to explain who s/he is/was and their achievements.		
**Lesson Plan**		
**Time**	**Activity**	**Resources/References**
24 min<br>(2-3 min/person)	Iceberg Icebreaker	W. Kamau Bell: What every American needs to know about White supremacy[10]<br>https://www.cnn.com/2020/07/19/opinions/united-shades-white-supremacy-kamau-bell/index.html<br><br>Displayed an image of the racism iceberg, and asked people to share a personal experience that relates to one of the components of the racism-iceberg.<br><br>Do you have any questions about anything on the iceberg and its relationship to racism?

30 min<br>(5 min/person)	Flipped Classroom: African American History	Students picked one article from The 1619 Project and shared what they learned with the group
10 min	The African Diaspora	Change your channel | Mallence Bart-Williams | TEDxBerlinSalon[11]<br>https://www.youtube.com/watch?v=AfnruW7yERA<br><br>The African Diaspora-What is it?[12]<br>https://www.yukonyouth.com/the-african-diaspora-what-is-it/<br><br>Experience Africa[13]<br>http://www.experience-africa.de/index.php?en_the-african-diaspora
10 min	Experiences with the Labor Movement	A Brief History of Labor, Race and Solidarity[14]<br>https://racial-justice.aflcio.org/blog/est-aliquid-se-ipsum-flagitiosum-etiamsi-nulla<br><br>African Americans and the American Labor Movement[15]<br>https://www.archives.gov/publications/prologue/1997/summer/american-labor-movement.html<br><br>Booker T. Washington on African Americans and Unions[16]<br>https://www.theatlantic.com/magazine/archive/1913/06/the-negro-and-the-labor-unions/529524/<br><br>How Black Activists Shaped the Labor Movement[17]<br>https://www.teenvogue.com/story/black-activists-shaped-the-labor-movement
16 min (1-2 min/person)	Share your Black Scientist s/hero	The enslaved man, Onesimus, and the smallpox “vaccine” - “All Sorts of People” (National Center for Case Study

		Teaching in Science)[18] https://sciencecases.lib.buffalo.edu/collection/detail.html?case_id=1065&id=1065<br><br>Matthew Henson and the first person to reach the North Pole (Stealing the Glory, by Pam Fraser Solomon)[19] https://www.matthewhenson.com/BBCnews.htm

Supplementary Note 3. Discussion on “Having courageous conversations (Braided narrative)”

Major objective: Understanding our personal relationship to white supremacy and anti-Black racism.

Learning Objectives:

1. Define and recognize white supremacy.
2. Gain the understanding that we all have a relationship with white supremacy, and to learn to identify the intersectionality of our experiences.
3. Acknowledge and accept past behavior and learn to move forward.

Supplementary Table 3. Pre-class assignment and lesson plan with time dedicated to each activity along with resources and references for Discussion 3.

**Part 1: Pre-class Assignment**		
None		
**Lesson Plan**		
**Time**	**Activity/Topic**	**Resources/References**
15 min	Introduction to Courageous Conversations format (see below)	Adapted from Courageous Conversations.[20] https://courageousconversation.com/
15 min	Read braided narrative of facilitators (see below)	Facilitator’s narrative (see below)
40 min	Write a personal narrative that addresses the prompt, “How did I learn the meaning of Blackness?”	Refer to slides (see below)
20 min	Break into groups and share/begin to braid your narratives.	

**Part 2: Pre-class Assignment**
Complete braiding your narrative with your group members

Lesson Plan		
**Time**	**Activity**	**Resources/References**
5 min	Refresher of purpose of conversations	Refer to slides (see below)
TBD by size of group	Read braided narrative of all groups	Allow time for discussion between groups
30 min	Whole group debrief/ reflection	

Presentation - Introduction to Courageous Conversations

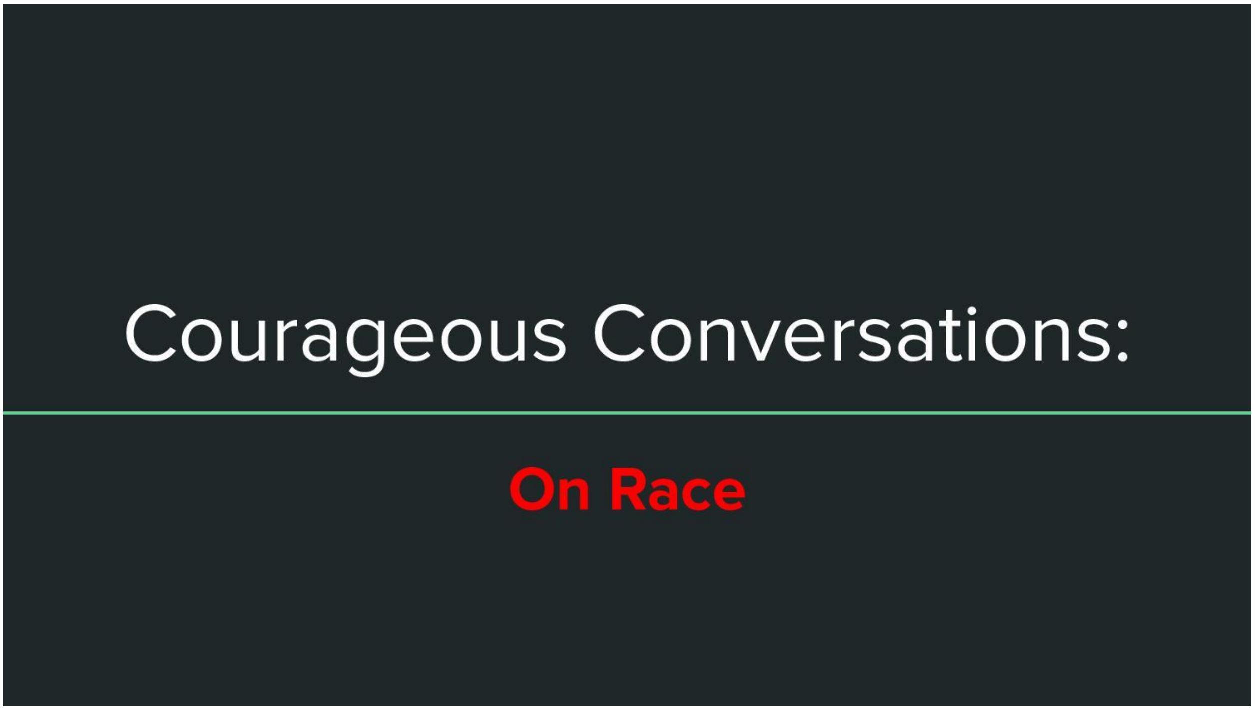

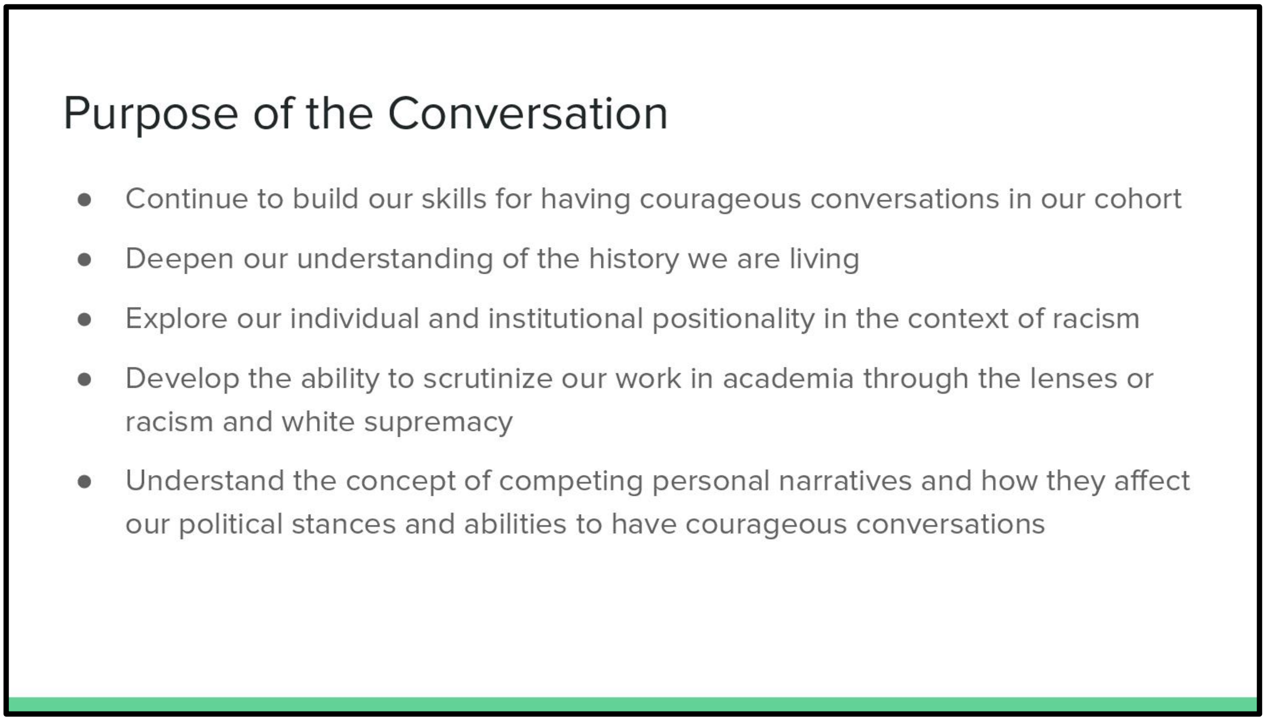

Norms and Agreements for Courageous Conversations

Singleton, G. 2005, Courageous Conversations on Race

- **Stay engaged**: “remaining morally, emotionally, intellectually, and socially involved in the dialogue.”
- **Experience discomfort**: discomfort in dialogue about race is inevitable
- **Speak your truth**: be open about your thoughts and feelings; do not just say what you think others want to hear
- **Expect and accept non-closure**: “hang out in uncertainty;” do not rush to quick solutions
- **We Don’t Rank Oppressions**: Injustice is injustice and it’s all wrong. We don’t compare our pains.

Some definitions

Race: a socially constructed characterization of individuals based on skin color and facial phenotypes

Racism: Any act that even unwittingly tolerates, accepts, or reinforces racially unequal opportunities or outcomes for people based on the social construct of race

Race privilege: A right or advantage that is given to some people and not others based on race

“Whiteness”: The component of each and every one of ourselves that expects assimilation to the dominant culture

White supremacy: the belief that white people are superior to those of all other races, especially the Black race, and should therefore dominate society

*Adapted from Singleton& Linton, 2006, Courageous Conversations about Race: A Field Guide for Achieving Equity in Schools

Managing Anxiety and Discomfort

- There is no way to get it right, because the injustices are so wrong. Be gentle with yourself if you fear “saying the wrong thing.”
- Being anti-racist requires not looking away from injustives that can cause anxiety and discomfort. Bearing witness and staying in the conversation is part of the work and also triggers different reactions based on positionality
- Avoid centering your anxiety and discomfort for others to address
- Know that everyone is an expert on their own experience
- We all inherited these injustices. We did not create them, What we do with them, in whatever vessel we occupy, is what matters.

Agenda

1. Opening questions
2. Prepared Braided Narratives
3. Write Your Narrative
4. Share Narrative in your Group
5. Reflections

Opening Questions

Please take a moment to jot down answers to the following questions:

What are you anxious about coming into this conversation?

What are you hoping for coming into this conversation?

Braided Narratives

How did I learn the meaning of Blackness?

Today:

1. Listen to the braided narrative that we will read aloud
2. Take 30 minutes write your own narrative
3. Break in groups and share/ Begin to braid your narratives

Next Class

1. Groups will share their narratives
2. Whole group debrief/ reflection

Example Braided Narrative

Jasmine

When I was two, I moved to Cary, NC, a small suburb in the Research Triangle Park area. Before starting kindergarten, I don't remember meeting another black child in my neighborhood. Being black did not bother me or the other children as far as I knew because no one even mentioned it. We all played together outside or at the neighborhood pool. My sister and I were welcomed in most homes, though a couple of our friends were never allowed to come to our house, their parents coming up with one excuse or another. It wasn't until I was 5 that another child actually pointed out the color of my skin. We were playing on the playground during my sister's tee-ball game and she asked me what my name was. When I said Jasmine, she paused and said, "Your name can't be Jasmine, you are not white." I told her that was my name, but she was welcome to call me something else, but I think she walked away.

When I started elementary school my neighborhood of a dozen or so white kids multiplied to hundreds. In my classroom, there was one other black kid. Everyone wanted him and I to be friends and get married and they talked about it all the time. In the first grade, I tested as Academically Gifted (AG) while many of my friends did not. This upset some of the neighborhood parents and my friend group got a little smaller. I didn't understand why people would be upset that I was in AG but I had a feeling it was because I was black. It was the only thing that made me different from any other kid in AG. In third grade, my parents pulled us out of our neighborhood school so we could go to a more racially diverse school in another city. I wasn't sad at all.

Rachel

I went to a predominantly Black elementary school until I was in the 5th grade, when my family moved expressly to get me and my sister into a better school district. We only moved about 20 minutes away but everything felt completely different at my new school. From then on, my classmates and friends were almost all white.

The hardest part about moving was missing my best friend, Ashley. My mother used to make fun of my grandparents for being concerned about me spending so much time with Ashley and especially about me spending time at her house. Learning that my family didn't approve of Ashley solely because she was Black was how I first came to understand anti-Blackness.

Throughout middle school and high school, I don't remember having any real awareness about the experiences of my Black classmates. I do remember that when we all started applying to colleges there was this perception that it was easier for Black students and for POC in general to get into college and to receive scholarships.

Saman

All through grade school, I went to a small Islamic school in Texas. Roughly half the students were of Arab origin, and the other half was south-asian. There were a few black families that had immigrated from Africa, and I saw them as other muslim people, just as different from the average "american" as me. I didn't think their experience as a POC in the US was any different than mine, just a slightly different color. The one thing that united us all was our religion, and we were taught to love each other as brothers and sisters in Islam and on this earth. When we learned in history class about the slavery in the US, the civil war, and the civil rights movement,

I was in awe of the strength of the people involved, and glad that that wasn't what it was like anymore.

Jasmine

For the rest of elementary, middle, and high school, I went to schools 30 minutes away so that I could be in a more diverse setting. But as I progressed through honors and then AP classes, I was often the only black student in class. My blackness was reduced by my teachers and peers to that of a token. I was often invited to represent the school in photo-ops and at different events, my fixed smile often bobbing in a sea of whiteness. These opportunities to "represent" black people in a positive light were juxtaposed with a constant stream of racist incidents perpetuated by teachers and students alike. For example, I was often discouraged by the guidance counselor from taking AP classes, deeming them "too difficult" for me. I was lucky to have parents that would constantly tell me my worth and what I was capable of, signing course approval forms, or going down to the principal's office if necessary. My blackness through education was always measured against whiteness.

Rachel

This idea that academic opportunities come more easily for Black people is a common theme I first picked up on in high school and now find laughable. It diminishes all that Black students are up against and has made me realize that Blackness means having your intelligence, abilities, and very presence constantly called into question every step of the way along your path.

All the time, I am watching my Black colleagues in academia endure this treatment with grace and humor in public. Only in private conversations do I hear confessions about how exhausting it is to cope with. Because Blackness also means hiding your pain to make other people feel more comfortable.

In college, I had trouble fitting in and sometimes felt very ostracized within my majority white peer groups. I discovered that among Black students and more diverse groups there was an environment of openness, acceptance, and support. I developed deeper relationships with my Black peers, who showed me that Blackness means valuing and upholding community.

I took a Women's Studies class in college and was introduced to intersectional feminism. I was shocked and horrified by our readings and discussions about the experiences of Black women, historically and present day. So much of it was news to me and taught me that Blackness also means resilience.

Saman

When 9/11 happened, our religion became the reason why we felt everyone else hated us. Every act of hate and prejudice was etched into our minds, and shaped how we viewed the world around us. I felt fear every time I went to an airport, every time someone looked at me with anger at my school uniform, which included a hijab, the islamic headcovering. Waves of antimuslim hate would ebb and flow, the cycle aligning with election years.

I left the bubble of my Islamic school to go away for college. I had a favorite hoodie, and one of my "friends" joked that I must carry a bomb around inside it, and I was legitimately terrified that the FBI was going to arrest me, as they had done to family members of my schoolmates. This is

why when I saw or heard about acts of prejudice and racism towards black people, I felt that I could understand. I had suffered too. I had seen other people like me suffer too.

Rachel

I recognized anti-Blackness at one of my first jobs in a biomedical research lab. Baffling things happened to my Black colleague and friend, like when our PI asked to take a picture of her hair while she was having a one-on-one meeting with him. But there were subtler ways in which she was consistently singled out and scrutinized. She had the same title as me, yet she was always being chastised while I was being praised for doing essentially the same work.

In graduate school, I saw more of the same. One of my Black colleagues was repeatedly and openly insulted by a white colleague, but I only ever heard my Black colleague characterized as being the one with the “attitude problem” by our PI.

Perhaps the most disturbing thing I’ve encountered time and time again at school and at work is the general attitude towards diversity funding. I have heard behind closed doors countless remarks insinuating that diversity funding is easier to obtain, which is why PIs often try to stretch the definition of “diverse” to justify having their white students and employees apply for it. The people who behave this way seem to be thinking of Blackness as an opportunity.

Saman

I made friends with someone in graduate school, who I sat with every day in class. There were two black girls in our year, and they always sat together too. My friend one day said out of the blue, they know they’re both here because of diversity, they wouldn’t have gotten in otherwise. I asked her how she knew, and she said, I mean… it’s obvious. Was it obvious? I myself was funded on an institutional DEI scholarship my program director nominated me for after I matriculated. Did my friend think I deserved to be here? This same friend made some nasty comments when we drove past a subsidized neighborhood, and I couldn’t believe I didn’t know that one of my closest friends of 3+ years was racist. But I was her friend, how could she be racist if I was her friend? I didn’t have much of a voice back then. I gently challenged her thoughts. She told me I didn’t know what I was talking about since I went to school with all immigrant families. I drifted away from her.

The distinction between anti muslim-hate and prejudice against black people hit me during a discussion with a couple of my new muslim friends I made in graduate school. We were talking about how every now and then in history, there are waves of prejudice against a certain group, Jews, Asians, Muslims, etc. What my friend said will always stay with me: no one has suffered the way black people have in America, they were enslaved and brought here, and have suffered from that enslavement ever since. I remembered my first friend from the beginning of graduate school. After that, the way I looked at the world changed. I noticed the way black people and white people were talked about on the news for the same crimes. If I noticed I was the only south-asian in the room, I also noticed if there was only one black person in the room. When Obama was elected president the first time, I had felt like this was a win for me, and people that were not white, like me. When he was reelected, I knew that it was much much more powerful for African Americans in this country.

Jasmine

I come from a large extended family that has many different shades of brown. We are a big and loud family that are quick to laugh and even quicker to love. My grandma raised my mom and my aunt in Iowa and still lives in the rural mid-West. From childhood, we saw pictures of my grandma with a large afro and pan-African clothes and heard rumors that she was once a black panther. My grandparents, parents, aunts, and uncles were all unapologetically beautiful, black, and excellent. I learned the importance of always working hard and striving for excellence so that no one could ever question whether I deserved the things that I achieved in life. More importantly, I learned that whatever I had or earned, I needed to use to help and serve others.

Saman

Before, I could see how Muslims were painted as terrorists using bright loud colors, in your face so you couldn't miss it, and so I could see the same about how black people were painted as bad and less worthy, but only in the bright loud colors. I started to see how they were painted that way in subtle colors, with the lightest touch.

Jasmine

In the early 70’s my grandma became a Baha’i. The Baha’i Faith teaches that we are all equal and unity in diversity is celebrated, but also refers to Black people as the “pupil of the eye” through which “the light of the spirit shineth forth”. Being raised a Baha’i, I was always around a diverse group of people and my blackness was cherished. I learned that everyone was created noble and to see everyone as a member of my family, and to let my heart burn with love and kindness for everyone. This outlook on life has helped me get through many tough times when my blackness has not been seen as a thing of beauty or excellence by others. I know that those people are wrong, I have hope that they will learn, and I strive to love them all the same.

Saman

I applied to a postdoctoral program where I had the opportunity to teach at HBCUs, something I was excited about. I took a class called how to teach with my cohort, and the professor wanted to include some social justice topics surrounding the abuse of black bodies in football. Soon after, the pandemic happened, the incident surrounding George Floyd happened, and several discussions about how our professors and leaders in our university had tokenized and committed microaggressions towards black trainees occurred. During these discussions, it was revealed that at least one clear microaggression happened in our class, and it seemed none of the non-black postdocs, including me, noticed. I still have a lot to learn. What happened, and is continuing to happen afterwards is amazing. We are all opening our minds up to learn about other experiences and how to be better people, and better brother and sisters to each other. We are learning together what Blackness is, and we will continue to learn. I had learned what blackness was in relation to racism. In observing my students, young black women at Spelman college, thriving in an environment that is entirely their own, I am learning about what blackness is in relation to community and sisterhood. I am hopeful for the future.

Rachel

I am optimistic that non-Black people are currently experiencing a collective shift in the way we view Blackness. I am very optimistic that we are headed towards a future where Blackness is celebrated outright. But we still have a long way to go.

Supplementary Note 4. Discussion on "Evidence-based practices for mentoring and supporting Black, Indigenous, and people of color (BIPOC) in STEM"

Major objective: Evaluating evidence-based practices for mentoring and increasing the retention of BIPOC in STEM.

Learning Objectives:

1. Identify the historical and social constructs that impede scientific success of BIPOC at various levels of education.
2. Evaluate current programs that attempt to address these inequities at various levels of education.
3. Brainstorm ways in which to improve programs that improve retention and representation of BIPOC in STEM at the undergraduate level.
4. Design a framework from which to foster inclusivity in the classroom and pass on (academic) cultural capital to our undergraduate students.

Supplementary Table 4. Pre-class assignment and lesson plan with time dedicated to each activity along with resources and references for Discussion 4.

**Pre-class Assignment**		
1. Read the abstracts (at minimum) of two assigned papers (see Table S5).<br>2. Find and read the abstract of a relevant paper.<br>3. Upload a copy of your paper to Google Drive.		
**Lesson Plan**		
**Time**	**Activity/Topic**	**Resources/References**
20 min	Have pairs explain their paper to each other, and the papers that they found<br><br>Guiding questions can include:<br>● Which level(s) of education is addressed in the assigned articles?<br>● What were the main conclusions of the assigned articles?<br>● How might you incorporate those conclusion(s) into your own teaching?<br>● How were the two articles different?	Papers can be found by the facilitators and/or brainstormed as a group prior to this discussion.<br><br>*Note to facilitators*: Pre-assign pairs to target their reading. We found this helped participants come prepared for the activity and made the activity run smoothly.

	• What new article did you find?<br>• What is the main conclusion of the article?<br>• What surprised you or was new to you?	
30 min	Recombine participants in groups where each individual represents a different education level, have everyone explain their findings (from the previous discussion), then discuss the learning objectives.<br><br>Guiding questions can include:<br>• Which level(s) of education is addressed in your articles?<br>• What were the main conclusions of the articles?<br>• Were any themes/ideas common among all the articles?<br>• What did you find most interesting or surprising?<br>• What will you incorporate into your teaching?	*Note to facilitators*: This activity is commonly called a 'jigsaw' activity, where teams discuss a similar topic and then recombine to discuss different aspects of the same topic.
Remaining Time	Debrief and ask the participants to reflect on and share what they learned. Identify any common themes.	*Note to the facilitators*: Here, it may be beneficial to discuss specific action items that you can take within your own institution to affect change, e.g., graduate student or faculty recruitment, outreach to local elementary schools, etc.
**Follow-Up Reflection Session**		
60 min<br>(entire class meeting)	Invite guest speakers to the class to discuss their personal experiences and strategies for incorporating evidence-based	*Note to facilitators*: Here, if possible, ensure that speakers are properly compensated for their time and effort in speaking about challenges that BIPOC

	practices to mentor and support Black, Indigenous, and people of color in their research and learning communities.	face in academia. This is often a thankless task, and even a small honorarium can signify a value and respect for their experiences.

Supplementary Table 5. Compiled list of suggested papers and associated topics pertaining to STEM education at different levels of education in the United States.

**Student Education Level**	**Suggested Readings/Papers**
K-12<br><br>Associated Topics<br>1. Outreach<br>2. Public vs. private school system<br>3. Pipeline<br>4. Family involvement	K-12 Diversity Pathway Programs in the E-STEM Fields: A Review of Existing Programs and Summary of Unmet Needs.[21]<br>https://www.jstem.org/jstem/index.php/JSTEM/article/view/2324<br><br>STEM Integration in K-12 Education.[22]<br>https://www.nap.edu/catalog/18612/stem-integration-in-k-12-education-status-prospects-and-an<br><br>A Scientist's Guide to Achieving Broader Impacts through K–12 STEM Collaboration.[23]<br>DOI: https://doi.org/10.1093/biosci/biu222
Undergraduate<br><br>Associated Topics<br>1. Inclusive teaching<br>2. Resilience<br>3. Community building<br>4. Cultural capital	Wilson-Kennedy et al. (2018) Transforming STEM Education through Collaborative Leadership at Historically Black Colleges and Universities. CBE - Life Sciences Education.[24]<br>DOI: 10.1187/cbe.18-06-0088<br><br>McGlynn, T.P. (2017) Identity Matters: Communicating About Equity and Opportunity for Students in Minority-Serving Institutions. Annals of the Entomological Society of America.[25]<br>DOI: https://doi.org/10.1093/aesa/sax050
Graduate<br><br>Associated Topics<br>1. Bridge programs<br>2. Resilience<br>3. Cultural capital<br>4. Diversity, equity, and inclusion (DEI) initiatives<br>5. Faculty composition	Wilkins-Yel et al. (2019) Linking intersectional invisibility and hypervisibility to experiences of microaggressions among graduate women of color in STEM.[26]<br>DOI: https://doi.org/10.1016/j.jvb.2018.10.018<br><br>Tseng et al. (2020) Strategies and support for Black, Indigenous, and people of colour in ecology and evolutionary biology. Nature Ecology and Evolution.[27]<br>DOI: https://doi.org/10.1038/s41559-020-1252-0<br><br>Porter et al. (2018) Burdens and benefits of diversity work: emotion management in STEM doctoral students. Studies in Graduate and Postdoctoral Education.[28]<br>DOI: https://doi.org/10.1108/SGPE-D-17-00041

Postdoctoral/Faculty<br><br>Associated Topics:<br>1. Mentorship<br>2. Funding<br>3. The facade of “fit”	Hofstra et al. (2020) The Diversity-Innovation Paradox in Science. PNAS.[29]<br>DOI: https://doi.org/10.1073/pnas.1915378117<br><br>White-Lewis, D.K. (2020) The Facade of Fit in Faculty Search Processes. CBE - Life Sciences Education.[30]<br>DOI:<br>https://doi.org/10.1080/00221546.2020.1775058<br><br>Zambrana et al. (2015) “Don’t Leave Us Behind”: The Importance of Mentoring for Underrepresented Minority Faculty. American Educational Research Journal.[31]<br>DOI: https://doi.org/10.3102/0002831214563063<br><br>Gumpertz et al. (2017) Retention and promotion of women and underrepresented minority faculty in science and engineering at four large land grant institutions. PLoS One.[32]<br>DOI: https://doi.org/10.1371/journal.pone.0187285<br><br>Glock et al. (2019) Teachers' attitudes towards ethnic minority students: Effects of schools' cultural diversity. Br J Educ Psychol.[33]<br>DOI: https://doi.org/10.1111/bjep.12248

Supplementary Note 5. Discussion on "Investigating microaggressions and macroaggressions as well as their impact in the classroom and beyond"

Major objective: Differentiate between microaggression and macroaggression and become familiar with their different forms or types.

Learning Objectives:
1. Become familiar with the different categories of racial microaggressions.
2. Quickly recognize and address microaggression when you witness or cause one.
3. Appropriately respond to a microaggression and make "microinterventions"
4. Articulate the impact of repeated (lifetime) of macro- and microaggressions on behavior, growth and achievement of students and colleagues.

Supplementary Table 6. Pre-class assignment and lesson plan with time dedicated to each activity along with resources and references for Discussion 5.

**Pre-class Assignment**		
None required although reading Sue et al. 2019[34] may be helpful.		
**Lesson Plan**		
**Time**	**Activity/Topic**	**Resources/References**
10 min	Work in small groups to discuss the following:<br>● Definition of microaggressions and macroaggressions without looking up the definition online<br>● How microaggressions are different from everyday rudeness and incivilities?<br><br>Each small group is also given an additional word to define: microassaults, microinsults, and microinvalidations.	Sue et al. 2007[35]
15 min<br>(5 min/group)	Each person gives at least one word to create a word cloud on microaggressions.<br><br>Discussion of the definition of microaggression and macroaggression.	Use Polleverywhere.com to create word cloud.

	Each group shares their definitions for subtypes of microaggressions with everyone.	
15 min	Work in small groups. Listen to scenarios (https://youtu.be/sLeRjqv5C1o)[36], identify the microaggression, and provide a 'gut reaction'. How would you respond as a bystander? Reflect on strategies for interventions.	Sue et al. 2019[34]<br><br>3 minute video provides 4 scenarios[36]: https://youtu.be/sLeRjqv5C1o
10 min	Debrief together on scenarios and discuss intervention options as a bystander.	Sue et al. 2019[34]
15 min	Each person provides one word for the impact of microaggression on the people of color individually and as a group.<br><br>Pose an open discussion question:<br>Do you think micro- and macroaggressions have an impact on the retention of students and faculty of color in academia?	Use Polleverywhere.com to create word cloud.<br><br>Sue et al. 2019[34]

Supplementary Note 6. Discussion on “Implementing social justice practices”

Major objective: Implementing social justice practices in higher education curriculum.

Learning Objectives:

1. Discuss how recent movements (e.g., #BlackInTheIvory, #BlackAFInSTEM, #PublishingPaidMe) have spurred more awareness about barriers BIPOC scientists and writers face.
2. Evaluate methods for implementing anti-racist practices in our labs, classrooms, departments, and colleges.
3. Brainstorm ways in which to increase transparency in negotiation proceedings, hiring practices, etc. in higher ed (e.g., Future PI Slack and other networks).

Supplementary Table 7. Pre-class assignment and lesson plan with time dedicated to each activity along with resources and references for Discussion 6.

**Pre-class Assignment**		
Draft a diversity, equity, and inclusion statement for your personal website, lab website, and/or course syllabus.		
**Lesson Plan**		
**Time**	**Activity/Topic**	**Resources/References**
10-15 min	Read/discuss articles about the #BlackInTheIvory, #BlackAFInSTEM, #PublishingPaidMe, #BlackinX movements and discuss how these efforts have increased awareness about barriers BIPOC scientists and writers face.	How #BlackInTheIvory put a spotlight on racism in academia[37] https://www.nature.com/articles/d41586-020-01741-7
30-40 min	Find resources to help you in your journey to becoming an anti-racist educator in the classroom and vocalize where/how you will openly acknowledge your commitment to diversity, equity, and inclusion (DEI).<br><br>Breakout into small groups (2-3 people) to peer review your partner’s DEI statement for	Becoming an anti-racist educator[38] https://wheatoncollege.edu/academics/special-projects-initiatives/center-for-collaborative-teaching-and-learning/anti-racist-educator/

	their personal website, lab website, and/or course syllabus.	
Remaining time	Discuss ways in which you can make your lab spaces, departments, universities more inclusive. What are existing programs? Where are initiatives falling short?<br><br>Lastly, make a list of ways in which your department/university could increase transparency in negotiation proceedings, hiring practices, etc. in higher ed. (e.g., incorporating rubrics into the interview process, telling graduate students/postdocs about existing Slack networks).	Chaudhary and Berhe (2020). Ten simple rules for building an antiracist lab PLoS Computational Biology.[39]<br>DOI: https://doi.org/10.1371/journal.pcbi.1008210)

Supplementary Note 7. Discussion on “Recognizing, alleviating and dismantling local systems of oppression”

Local systems of oppression like Institutional oppression refer to the systematic mistreatment of particular groups through established laws, customs or practices within a society. Systemic oppression is not always readily apparent; it may not appear as blatant racism, for example, but it does play a role in institutions such as education, health care, housing and criminal justice systems or even religion. However, as we talk about local systems of oppression, it is important to address individual as well as institutional responsibilities.

Major Objective: Recognize different forms of oppression and be familiar with ways of alleviating and dismantling local systems of oppression.

Learning Objectives:

1. **Recognizing oppression:** Explain the concepts and theories of oppression, i.e., “One Cannot Resolve What Is Not Acknowledged”.
    a. What is oppression?
    b. What is the reason for oppression?
    c. Psychological implications of oppression
    d. Define social justice, economic security and equality.
2. **Alleviating and Dismantling oppression:** How do we make the consequences of oppression less severe or how do we suppress it, i.e., “How Can One Resolve Once Acknowledgement Happens”.
    a. Identify the role(s) played by human behavior in the social environment.
    b. Explore personal biases and stereotypes that can affect human behavior.
    c. Identify the impact of privilege and oppression and the potential power dynamics of race in the context of how the dynamics of oppression impact the human developmental process.
    d. Identify action strategies used to address and dismantle oppression
    e. Identify ways individual, social movements and institutions can promote justice and equality and to alleviate oppression.

Supplementary Table 8. Pre-class assignment and lesson plan with time dedicated to each activity along with resources and references for Discussion 7.

**Pre-class Assignment**
1. Read your assigned article (see suggested list of articles below).<br>2. Make a discussion board post reflecting on your assigned article and consider the following questions as a guide:<br>a. What is oppression in the context of this article/incident?<br>b. Can you speculate on where this oppression originated?

c. What were the implications of this oppression on the victim? On the general public?<br>3. Comment on a classmate's reflection post.<br>4. Find and share a new article or resource with strategies to alleviate or dismantle systems of oppression.
**Lesson Plan**

Time	Activity/Topic	Resources/References
25 min	Have a conversation about the assigned readings	See potential list of articles below.<br><br>*Note to facilitators:* We encourage you to find your own articles that address systematic oppression.
1 hr	Have students share the articles and resources they found, what they learned from them, and have a broader discussion as a group.	

Possible articles for reflection:

- The 'Affluenza' Teen vs. the Value of Black Life[40] - https://www.theroot.com/the-affluenza-teen-vs-the-value-of-black-life-1790862202
- ‘Affluenza’ Case Highlights Socioeconomic, Racial Disparities in Justice[41] - https://jjie.org/2014/03/18/affluenza-case-highlights-socioeconomic-racial-disparities-in-justice/
- Public opinion on the affluenza defense, race, and sentencing decisions: results from a statewide poll[42] - https://www.tandfonline.com/doi/full/10.1080/0735648X.2015.1108550
- Story of mother sentenced to jail for enrolling child in different district resurfaced amid college scandal[43] - https://thehill.com/blogs/blog-briefing-room/news/434051-story-of-mother-sentenced-to-jail-for-enrolling-child-in
- Louisiana Supreme Court upholds Black man's life sentence for stealing hedge clippers more than 20 years ago[44] - https://www.cnn.com/2020/08/06/us/louisiana-supreme-court-trnd/index.html
- From 'BBQ Becky' to 'Golfcart Gail,' list of unnecessary 911 calls made on blacks continues to grow[41] - ‘Affluenza’ Case Highlights Socioeconomic, Racial Disparities in Justicehttps://abcnews.go.com/US/bbq-becky-golfcart-gail-list-unnecessary-911-calls/story?id=58584961

Supplementary Note 8. Discussion on "Fostering anti-racist behavior among our students, academic peers, and community organizations"

Major objective: Construct your action plan as an anti-racist in your family, department and classroom

Learning Objectives:

1. Define anti-racism.
2. Recognize why we need to take personal responsibility for eliminating racism.

Supplementary Table 9. Pre-class assignment and lesson plan with time dedicated to each activity along with resources and references for Discussion 8.

**Pre-class Assignment**		
None		
**Lesson Plan**		
**Time**	**Activity**	**Resources/References**
15 min	Brainstorm a definition of anti-racism as a group. Individuals contribute words or phrases to a word cloud that informs the definition.	Use Polleverywhere.com to create word cloud.
10 min	In small groups, come up with at least 3 action plan points on how to foster anti-racism in a classroom setting.	Example of an action plan[45]: https://www.monash.edu/cultural-inclusion/resources-and-support/an-anti-racist-culture
15-20 min	Each small group shares with everyone and a group action plan that all agree on is outlined.	Use Google Docs so that everyone can have a copy of the action plan.
5 min	Debrief	

References

1. Crum P. Your Reality Might Not be Mine: Sensory Perception and Empathy - TEDxLA. https://www.youtube.com/watch?v=SYytiQmXNTc (2017).

2. Kemp A. How to Have a Voice and Lean Into Conversations About Race - TEDxWilmington. https://www.youtube.com/watch?v=IF--2vGj7Tg (2017).

3. Vera A, Ly LL. White woman who called police on a black man bird-watching in Central Park has been fired. CNN (2020).

4. NorQuestVids. Incident A8 An Uncomfortable Conversation. https://www.youtube.com/watch?v=5-3gbex09tk (2016).

5. NorQuestVids. Incident D19 Its Clearly Language Thing. https://www.youtube.com/watch?v=29bobQDLrBA (2016).

6. NorQuestVids. Incident D13 Coming Up Against Hard Edges. https://www.youtube.com/watch?v=foFdqmdiaR0 (2016).

7. NorQuestVids. Incident A11 So Were All in Agreement Then. https://www.youtube.com/watch?v=6sbNk4cEYbY (2016).

8. Aduayom D, Hannah-Jones N, Mhute W. The 1619 Project. *The New York Times*. https://www.nytimes.com/interactive/2019/08/14/magazine/1619-america-slavery.html (2019).

9. Glaude Jr E. The Lie That Underpins All Injustices Facing Black Americans | NowThis. https://www.youtube.com/watch?app=desktop&v=I205IQw6ns8 (2020).

10. Bell WK. What every American needs to know about White supremacy. *CNN*. https://www.cnn.com/2020/07/19/opinions/united-shades-white-supremacy-kamau-bell/index.html (2020).

11. Bart-Williams M. Change your channel | Mallence Bart-Williams | TEDxBerlinSalon. https://www.youtube.com/watch?app=desktop&v=AfnruW7yERA (2015).

12. Adamek T. The African Diaspora-What is it? In: *Yukon Youth* https://www.yukonyouth.com/the-african-diaspora-what-is-it/ (2018).

13. The African Diaspora. *The Institute for Cultural Diplomacy Inc.* https://www.experience-africa.de/index.php?en_the-african-diaspora (2021).

14. A Brief History of Labor, Race and Solidarity. *Labor Commission on Racial and Economic Justice*. https://racial-justice.aflcio.org/blog/est-aliquid-se-ipsum-flagitiosum-etiamsi-nulla (2017).

15. Cassedy JG. African Americans and the American Labor Movement. In: *Federal Records and African American History* https://www.archives.gov/publications/prologue/1997/summer/american-labor-movement.html (1997).

16. Washington BT. The Negro and the Labor Unions. In: *The Atlantic* (1913).

17. Kelly K. Black Civil Rights Activists Were Crucial to the Labor Movement. In: *Teen Vogue* (2019).

18. Aguirre KM. All Sorts of People. https://sciencecases.lib.buffalo.edu/collection/detail.html?case_id=1065&id=1065.

19. Solomon PF. Stealing the Glory, by Pam Fraser Solomon. https://www.matthewhenson.com/BBCnews.htm (2021).

20. Courageous Conversation - Providing Training, Coaching and Consulting for Millions of Racial Equity Leaders Around the World. *@CCAboutRace*. https://courageousconversation.com/ (2021).

21. Cagle LC, Rosina G, Nicolette. K-12 Diversity Pathway Programs in the E-STEM Fields: A Review of Existing Programs and Summary of Unmet Needs. *https://wwwjstemorg/jstem/indexphp/JSTEM*, (2018).

22. Engineering NAo, Council NR. *STEM Integration in K-12 Education: Status, Prospects, and an Agenda for Research*. The National Academies Press (2014).

23. Komoroske LM, Hameed SO, Szoboszlai AI, Newsom AJ, Williams SL. A Scientist's Guide to Achieving Broader Impacts through K–12 STEM Collaboration. *BioScience* **65**, 313-322 (2015).

24. Wilson-Kennedy ZS, Kanipes MI, Byrd GS. Transforming STEM Education through Collaborative Leadership at Historically Black Colleges and Universities. *CBE—Life Sciences Education* **17**, es13 (2018).

25. McGlynn TP. Identity Matters: Communicating About Equity and Opportunity for Students in Minority-Serving Institutions. *Annals of the Entomological Society of America* **110**, 480-483 (2017).

26. Wilkins-Yel KG, Hyman J, Zounlome NOO. Linking intersectional invisibility and hypervisibility to experiences of microaggressions among graduate women of color in STEM. *Journal of Vocational Behavior* **113**, 51-61 (2019).

27. Tseng M, El-Sabaawi RW, Kantar MB, Pantel JH, Srivastava DS, Ware JL. Strategies and support for Black, Indigenous, and people of colour in ecology and evolutionary biology. *Nature Ecology & Evolution* **4**, 1288-1290 (2020).

28. Porter KB, Posselt JR, Reyes K, Slay KE, Kamimura A. Burdens and benefits of diversity work: emotion management in STEM doctoral students. *Studies in Graduate and Postdoctoral Education* **9**, 127-143 (2018).

29. Hofstra B, Kulkarni VV, Munoz-Najar Galvez S, He B, Jurafsky D, McFarland DA. The Diversity–Innovation Paradox in Science. *Proceedings of the National Academy of Sciences* **117**, 9284 (2020).

30. White-Lewis DK. The Facade of Fit in Faculty Search Processes. *The Journal of Higher Education* **91**, 833-857 (2020).

31. Zambrana RE, Ray R, Espino MM, Castro C, Douthirt Cohen B, Eliason J. "Don't Leave Us Behind": The Importance of Mentoring for Underrepresented Minority Faculty. *American Educational Research Journal* **52**, 40-72 (2015).

32. Gumpertz M, Durodoye R, Griffith E, Wilson A. Retention and promotion of women and underrepresented minority faculty in science and engineering at four large land grant institutions. *PLOS ONE* **12**, e0187285 (2017).

33. Glock S, Kovacs C, Pit-ten Cate I. Teachers' attitudes towards ethnic minority students: Effects of schools' cultural diversity. *British Journal of Educational Psychology* **89**, 616-634 (2019).

34. Sue DW, Alsaidi S, Awad MN, Glaeser E, Calle CZ, Mendez N. Disarming racial microaggressions: Microintervention strategies for targets, White allies, and bystanders. *Am Psychol* **74**, 128-142 (2019).

35. Sue DW*, et al.* Racial microaggressions in everyday life: implications for clinical practice. *Am Psychol* **62**, 271-286 (2007).

36. Majewska AA. Microaggression Scenarios. https://youtu.be/sLeRjqv5C1o (2021).

37. Subbaraman N. How #BlackInTheIvory put a spotlight on racism in academia. *Nature* **582**, 327 (2020).

38. Becoming an Anti-Racist Educator. *Wheaton College Massachusetts*. https://wheatoncollege.edu/academics/special-projects-initiatives/center-for-collaborative-teaching-and-learning/anti-racist-educator/ (2021).

39. Chaudhary VB, Berhe AA. Ten simple rules for building an antiracist lab. *PLOS Computational Biology* **16**, e1008210 (2020).

40. Crockett Jr. SA. The 'Affluenza' Teen vs. the Value of Black Life. TheRoot (2021).

41. Zhu A. 'Affluenza' Case Highlights Socioeconomic, Racial Disparities in Justice. In: *Juvenile Justice Information Exchange*. @jjienews (2014).

42. Douds AS, Howard D, Hummer D, Gabbidon SL. Public opinion on the affluenza defense, race, and sentencing decisions: results from a statewide poll. *Journal of Crime and Justice* **39**, 230-242 (2016).

43. Daugherty O. Story of mother sentenced to jail for enrolling child in different district resurfaced amid college scandal. In: *The Hill* https://thehill.com/blogs/blog-briefing-room/news/434051-story-of-mother-sentenced-to-jail-for-enrolling-child-in (2019).

44. Kay J, Leah Asmelash CNN. Louisiana Supreme Court upholds Black man's life sentence for stealing hedge clippers more than 20 years ago. In: *CNN* (2021).

45. Fostering an anti-racist campus culture. *Monash University*. https://www.monash.edu/cultural-inclusion/resources-and-support/an-anti-racist-culture (2021).
